# Endogenous Sex Steroid Hormones, Sex Hormone-Binding Globulin, and Risk of All-Cause and Cause-Specific Mortality: A Systematic Review and Dose-Response Meta-Analysis of Prospective Cohort Studies

**DOI:** 10.1210/clinem/dgaf262

**Published:** 2025-05-07

**Authors:** Hamidreza Raeisi-Dehkordi, Mojgan Amiri, Sara Beigrezaei, Hugo G Quezada-Pinedo, Farnaz Khatami, Fadi Alijla, Marinka Steur, Beatrice Minder, Angeline Chatelan, Trudy Voortman, Yvonne T van der Schouw, Oscar H Franco, Taulant Muka

**Affiliations:** Department of Global Public Health and Bioethics, Julius Center for Health Sciences and Primary Care, University Medical Center (UMC) Utrecht, Utrecht University, 3584 CG Utrecht, The Netherlands; Institute of Social and Preventive Medicine (ISPM), University of Bern, 3012 Bern, Switzerland; Department of Epidemiology, Erasmus MC, University Medical Center, 3000 CA Rotterdam, The Netherlands; Department of Global Public Health and Bioethics, Julius Center for Health Sciences and Primary Care, University Medical Center (UMC) Utrecht, Utrecht University, 3584 CG Utrecht, The Netherlands; The Generation R Study Group, Erasmus MC, University Medical Center Rotterdam, 3000 CA Rotterdam, The Netherlands; Institute of Social and Preventive Medicine (ISPM), University of Bern, 3012 Bern, Switzerland; Community Medicine Department, Tehran University of Medical Sciences, 1417613151 Tehran, Iran; Institute of Social and Preventive Medicine (ISPM), University of Bern, 3012 Bern, Switzerland; Department of Epidemiology, Erasmus MC, University Medical Center, 3000 CA Rotterdam, The Netherlands; Division of Human Nutrition and Health, Wageningen University & Research, 6708 PB Wageningen, The Netherlands; Public Health & Primary Care Library, University Library of Bern, University of Bern, 3012 Bern, Switzerland; Geneva School of Health Sciences, HES-SO University of Applied Sciences and Arts Western Switzerland, CH-1227 Geneva, Switzerland; Department of Epidemiology, Erasmus MC, University Medical Center, 3000 CA Rotterdam, The Netherlands; Meta-Research Innovation Center at Stanford (METRICS), Stanford University, Stanford, CA 94303, USA; Department of Global Public Health and Bioethics, Julius Center for Health Sciences and Primary Care, University Medical Center (UMC) Utrecht, Utrecht University, 3584 CG Utrecht, The Netherlands; Department of Global Public Health and Bioethics, Julius Center for Health Sciences and Primary Care, University Medical Center (UMC) Utrecht, Utrecht University, 3584 CG Utrecht, The Netherlands; Epistudia, 3008 Bern, Switzerland

**Keywords:** androgens, sex differences, testosterone, SHBG, estradiol, death

## Abstract

**Background:**

While abundant research suggests a sex-specific role of endogenous sex steroid hormones in chronic diseases, research on mortality remains inconclusive. We quantified the sex-specific associations of endogenous sex steroid hormones, including total testosterone (TT), free testosterone, bioavailable testosterone, estradiol, dehydroepiandrosterone (DHEA), and DHEA sulfate (DHEAS), and sex hormone-binding globulin (SHBG) with risk of all-cause and cause-specific mortality in the general population.

**Methods:**

Embase, Medline, Web of Science, and Cochrane Central were searched and population-based cohort studies investigating the association of interest were included. The risk of bias was assessed using the ROBINS-E tool. The certainty of evidence was evaluated using the GRADE framework. Pooled hazard ratios (HRs) and 95% CI were calculated using a random effects model for the top vs bottom tertile of sex hormones and risk of mortality.

**Results:**

The systematic review included 53 publications with 359 047 participants. A significant association was observed between higher level of TT and risk of all-cause mortality (HR [95% CI]: 0.89 [0.83-0.97], n = 19 studies) in men, while no association was found in women. Dose-response analysis suggested a significant U-shaped association between TT and all-cause mortality in men and a J-shaped association in women. Higher SHBG level was significantly associated with higher risk of all-cause mortality in women (1.25 [1.13-1.39], n = 3) and no association was observed in men. Additionally, higher DHEAS levels were associated with lower risk of all-cause mortality in men (0.72 [0.57-0.91], n = 6) and no association was observed in women.

**Conclusion:**

This meta-analysis reveals a dose-response link between endogenous sex steroid hormones and mortality, highlighting the need for sex-specific studies on hormone modulation's impact on mortality and longevity.

Abundant evidence suggests a role of endogenous sex steroid hormones in the development of chronic diseases and associated risk factors, which might be sex-specific (1). For instance, hypoandrogenism in men and hyperandrogenic conditions in women have been linked to obesity, insulin resistance, and incidence of type 2 diabetes and cardiovascular disease (CVD) (2-8).

While endogenous sex steroid hormones have implications for chronic diseases, findings on mortality are inconsistent. Several studies have shown an association between lower levels of total testosterone (TT) and higher risk of either all-cause or cause-specific mortality in men (9-11), while studies in women are indicative of either no association (12, 13) or higher risk of all-cause mortality (14). While the majority of studies showed no association between sex hormone-binding globulin (SHBG) and risk of all-cause and cause-specific mortality (10, 15-17), findings of a large population-based study reported a higher risk of all-cause mortality with higher level of SHBG in both men and women (14) and of cancer and CVD mortality in men. The association of estradiol (E2) and risk of mortality has been studied in men, and the findings are controversial particularly for all-cause and cancer mortality (10, 11). Some studies have found an association between E2 and risk of all-cause (11) and cause-specific mortality (18), others found no significant association with mortality risk (19). Similar controversy has been explored also with menopausal therapy, with the largest clinical trial showing no impact on mortality (20).

Additionally, some studies reported a protective role of dehydroepiandrosterone sulfate (DHEAS) against all-cause or CVD mortality in men (21, 22) and women (23); on the contrary, other studies found no association in either men (23, 24) or women (25, 26).

Although a few meta-analyses have been performed on the association of endogenous sex steroid hormones and risk of mortality; they only included studies on men, examined a single endogenous sex steroid hormone (mainly TT), and investigated the association only with all-cause mortality (27-29). Furthermore, none of these studies investigated the shape of association.

Therefore, we systematically summarized and quantified the available evidence on the sex-specific associations of endogenous sex steroid hormones, including TT, bioavailable testosterone (BT), free testosterone (FT), DHEAS, E2, and dehydroepiandrosterone (DHEA), as well as SHBG, with risk of all-cause and cause-specific mortality, including CVD, cancer, and other causes in the general population.

## Methods

This systematic review was designed and conducted in accordance with 2 recent systematic review guidelines and is reported following the PRISMA (Preferred Reporting Items for Systematic reviews and Meta-Analyses) recommendations (30-32). The study protocol was registered in the PROSPERO database in May 2022 (registration number: CRD42022329605).

### Data Sources and Search Strategy

We combined terms for endogenous sex steroid hormones and mortality-related keywords to find potentially relevant studies. Embase.com, Medline ALL (Ovid), Web of Science Core Collection, and Cochrane Central were searched until March 21, 2024. Furthermore, filters to exclude conference abstracts, case-report studies, non-adult populations, and animal studies were applied. The search strategy was developed by an expert research librarian (B.M.). The search results were imported in EndNote and de-duplicated with the method as described by Bramer et al (33). The details of the search strategy and keywords are presented in Supplementary Table S1 (34). To complete our search, the references of the included studies and published reviews were manually reviewed.

### Eligibility Criteria

We selected original published studies with aggregate data if (i) they had a prospective design (case-cohort, nested case control, and cohort studies); (ii) were conducted in a general adult population (≥18 years); (iii) measured at least one of the following endogenous sex steroid hormones as exposure at baseline: TT, BT, FT, DHEA, DHEAS, and E2 or SHBG; (iv) reported associations with all-cause or cause-specific mortality: CVD, cancer, and other causes; and (v) reported associations as adjusted effect estimates (relative risk, hazard ratio, or odds ratio) with 95% CI. Among the reported models, we included the one with the most adjustments. For publications reporting results from the same study and the same exposure and outcome, those with the largest number of participants, or the most recent publication, were included in the analysis; in case they had different publications on non-overlapping sex hormones and risk of mortality, we included the additional endogenous sex steroid hormones from the other publications conducted on the identical cohort.

Retrospective studies, conference abstracts, ecological studies, case reports, case-series, letters to the editor, conference proceedings, narrative reviews, systematic reviews, or meta-analyses as well as studies conducted in animals, children, or adolescents were excluded. Also, studies conducted solely on patients were excluded. We did not include non-English language articles.

### Study Selection and Data Extraction

All titles/abstracts were screened in duplicate by 2 independent groups of researchers (H.R.D., M.A., and S.B. together with H.Q., F.A., and F.K.H.) according to the eligibility criteria. Afterwards, all selected full-text records were reviewed again in duplicate (by H.R.D., M.A., and S.B.). The data from the included studies were extracted based on a predesigned form (by H.R.D., M.A., and S.B.) and both steps were conducted with EndNote 20. We extracted the first author's name, study design, study name, publication year, location, number of participants, sex distribution of the population, age, follow-up duration, level of sex steroid hormones, sex steroid hormones assessment method, number of deaths, causes of mortality, adjustments, and hazard/ risk/odds ratios.

### Risk of Bias Assessment and Certainty of Evidence

Risk of bias assessments were conducted in duplicate by independent reviewers (H.R.D., M.A., and S.B.) and disagreements were resolved by consensus. We evaluated the risk of bias of the cohort studies with the recent version of the ROBINS-E tool (Risk Of Bias In Non-randomized Studies—of Exposures) (35). The certainty of evidence was assessed for all associations with the updated Grading of Recommendations Assessment, Development, and Evaluation (GRADE) tool by investigators independently (H.R.D., M.A., and S.B.) (36). More information on these tools is provided in Supplementary Information 1 (34).

### Data Analysis

We used the DerSimonian-Laird random effects model to calculate pooled hazard ratios (HR) and 95% CIs (37). For analyses with a small number of studies (eg, ≤ 5), we applied the Hartung-Knapp method for random effects to calculate pooled HR and 95% CI (38). Relative risk was treated as HR and for studies reporting odds ratios (ORs), only if the OR was between 0.5 and 2.5, the values were included and treated as HR (39). We extracted the risk estimates of the most adjusted model and stratified by sex. To enable a consistent and standardized approach in the meta-analysis, all risk estimates for the association between endogenous sex steroid hormones and mortality were transformed to the top vs bottom third of sex hormones distribution in each study (40). Based on this method, we need to use conversion factors driving based on the ratio of expected differences in mean levels of the standardized exposure, for the target comparison vs reported comparison. Examples of the risk conversion methods are described in Supplementary Information 2 (34). For studies that used other categories than the first one as the reference category, we changed the reference category to the lowest one and recalculated the risk estimates using the method developed by Hamling (41). We calculated standard errors (SE) using a method developed by Greenland (42) for studies that did not report CI or SE. Heterogeneity between studies was assessed using the *I^2^* (43). We conducted subgroup analyses for each exposure and specific outcomes when 10 or more studies were included in the meta-analysis (44). Subgroup analyses were performed by: follow-up duration (<10 or ≥10 years), geographical region (Australia, Europe, North America), and whether studies adjusted for some risk factors such as alcohol consumption, lipid markers, blood pressure, physical activity, history of hypertension, type 2 diabetes, and hyperlipidemia. Sensitivity analyses were also performed to explore if the results were robust using leave-one-out analysis, excluding each study at a time from the analysis. Publication bias was assessed when at least 10 studies were available with Egger's test and by visually exploring funnel plots for asymmetry (45, 46). For the linear dose-response meta-analyses, we estimated summary HRs and 95% CI for each prospective cohort study according to the method developed by Crippa et al (47). Additionally, one stage weighted mixed effects meta-analysis was used to explore curvilinear dose-response associations (47). Further information on these analyses is explained in the supplements.

We performed the statistical analyses of our meta-analysis in R (Version 4.0.5) using meta, dmetar, and dosresmeta packages and Stata (Version 16.0). *P* < .05 was considered significant.

## Results

### Eligible Studies

**Figure 1.**
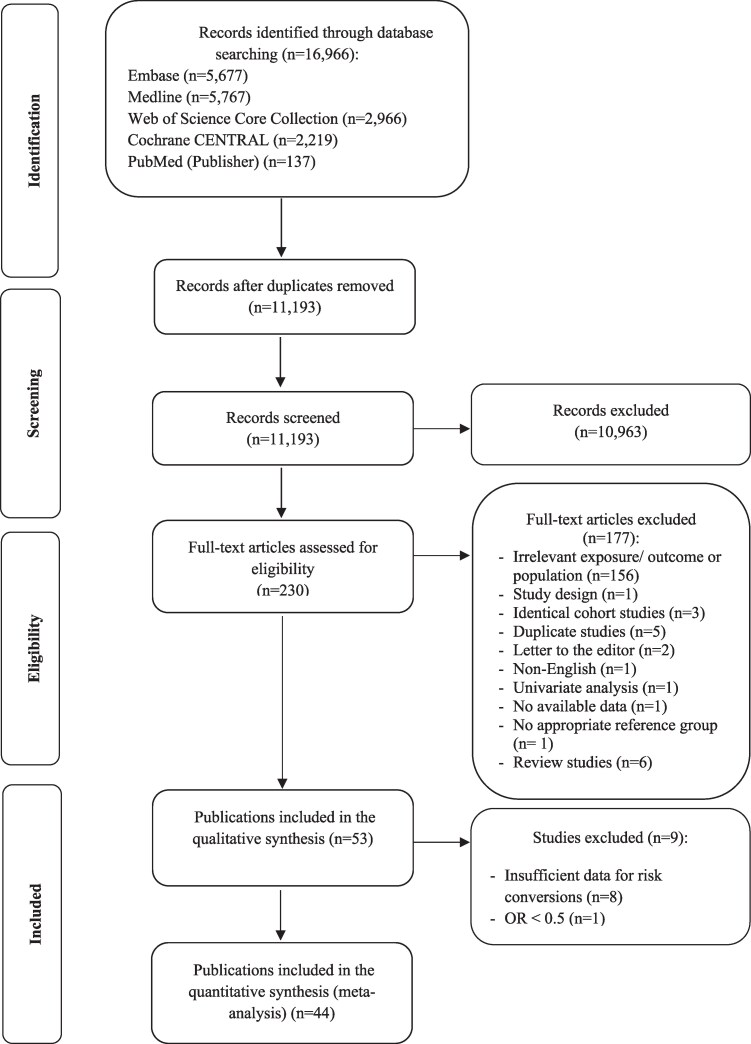
Study selection procedure.

Among 11 193 references, 230 full-text articles were assessed for eligibility. Of those, 177 references were excluded, leaving 53 publications, based on 34 unique studies, to be included in the qualitative analysis. Due to insufficient data to perform risk conversions (48-55) or an OR lower than 0.5 (56), 9 of these were not qualified to be part of the quantitative synthesis. Eventually, 44 publications, based on 28 studies, were included in the meta-analysis. Figure 1 represents the study selection procedure.

### Study Characteristics

Characteristics of the included publications are presented in more detail in Table 1. Briefly, the publication date varied between 1986 and 2024. Overall, there were 359 047 participants included and 28 212 all-cause mortality events, 7512 CVD deaths, 6895 cancer deaths, and 2550 death events due to the other causes. Among these studies, the median follow-up duration was 10 years with an interquartile range of 7.4 and 14.9 years. The majority of the studies were conducted in European countries (n = 28) followed by North American countries (n = 17), Australia (n = 6), and Asia (n = 2). All except 3 studies (57-59) reported results stratified by sex. The assessment methods for hormones and SHBG reported in each study are summarized in Supplementary Table S2 (34). Based on the ROBINS-E tool, 11 publications (20.7%) rated as low risk of bias, 16 (30.2%) as having some concerns, and 26 (49.05%) were judged to be at high risk of bias (Supplementary Table S3) (34).

**Table 1.** Characteristics of the included studies

Study characteristics					Participant characteristics			Exposure and outcomes					
									No. of deaths				
Author, year	Country	Study name	Design	Follow-up^*a*^	Sex	Age^*b*^	No. of participants	Sex hormones	All-cause	CVD	Cancer	Other	Adjustments
Araujo et al, 2007 (17)	USA	Massachusetts Male Aging Study	Prospective cohort	15.3	M	40-70	1686	TT, FT, SHBG	395	101	127	—	Age, BMI, WC, HDL, SBP, race (White vs Other), alcohol consumption, calories expended in PA, ever smoking, self-assessed health (excellent, very good, good, or fair/poor), self-reported chronic disease (heart disease, HTN, or DM)
Antonio et al, 2022 (60)	Multiple countries^*c*^	European Male Aging Study	Prospective cohort	12.6	M	59.4 ± 10.8	1788	SHBG, E2	420	—	—	—	Study Centre, BMI, alcohol intake, smoking status, PA, number of comorbidities at baseline
Appiah et al, 2022 (18)	USA	NHANES (1988-1991)	Prospective cohort	25.2	M	35.7 ± 11.6	954	E2	40	—	—	—	Age. Race, ethnicity, and education. BMI, smoking status, SBP, alcohol intake, PA, HDL, TC, CRP, TT
Barrett-Connor et al, 1995 (26)	USA	Rancho Bernardo Study	Prospective cohort	19	F	65.5	942	DHEAS	—	192	—	—	Age, cholesterol, BP, smoking, estrogen replacement therapy, obesity, FPG, family history of heart disease
Barrett-Connor et al, 1986 (22)	USA	Rancho Bernardo Study	Prospective cohort	12	M	50-79	208	DHEAS	65	38	—	—	Age, obesity, SBP, TC, FPG, current smoking status
Baylis et al, 2012 (59)	UK	Hertfordshire Ageing Study	Prospective cohort	10	B	65-70	254	TT, SHBG, DHEAS	NM	—	—	—	Age, gender, baseline height, weight for height, smoking, alcohol, social class and walking speed, baseline number of systems medicated as a marker of comorbidity
Belladelli et al, 2021 (19)	USA	NHANES (1999-2004)	Prospective cohort	9.5	M	43.1 ± 20.78	1109	E2	237	44	63	—	Age, race, education, BMI, smoking status, alcohol use, comorbidities
Benn et al, 2015 (52)	Denmark	Copenhagen City Heart Study	Nested case-control*^d^*	19	F	59 (49-65)	4716 (TT), 4600 (E2)	TT, E2	2716	659	531	—	Age, BMI, TC, HDL, menopausal status, age at menopause, HTN, DM, current smoking, alcohol intake
Cappola et al, 2009 (58)	USA	Cardiovascular Health Study	Prospective cohort	17	B	72.85 ± 5.41	950	DHEAS	NM	—	—	—	Age, race, cardiovascular disease, pulmonary disease, DM, cancer, depression
Chan et al, 2016 (61)	Australia	Busselton Health Study	Prospective cohort	14.9	M	50.3 ± 16.8	1804	TT, FT, DHEA, E2	319	141	—	—	Age, smoking, vigorous exercise, alcohol, BMI, DM, CVD history, COPD history, non-skin cancer history, SBP, HTN, lipids medication, cholesterol, HDL, TG, CRP, creatinine
Cummings-Vaughn et al, 2011 (62)	USA	NM	Prospective cohort	9	M	78.89 ± 6.9	190	TT, BT	41	—	—	—	Age, comorbidities (arthritis, HTN, cardiopulmonary conditions, cerebrovascular disease, DM, cancer [malignant], and kidney disease), lower body functional limitations, physician visits
Enomoto et al, 2008 (53)	Japan	Tanushimaru Study	Prospective cohort	27	M	51.1 ± 16.4	396	DHEAS	95	—	—	—	Age
Enomoto et al, 2008 (53)	Japan	Tanushimaru Study	Prospective cohort	27	F	50.4 ± 15.6	544	DHEAS	124	—	—	—	Age
Forti et al, 2012 (57)	Italy	Conselice Study of Brain Aging	Prospective cohort	8	B	74.19 ± 6.65	920	DHEAS	248	—	—	—	Age, sex, smoking, BMI, IHD, preexisting major diseases, disability, frailty, Geriatric Depression Scale, the Mini-Mental State Examination, high serum CRP
Feldman et al, 2000 (24)	USA	Massachusetts Male Aging Study	Prospective cohort	9	M	40-70	1167	DHEAS	—	22	—	—	Age
Guadalupe-Grau et al, 2017 (56)	Spain	Toledo Study for Healthy Aging	Prospective cohort	5.5	F	NM	796	TT, DHEA, DHEAS, E2	92	—	—	—	Age, PASE, hip and waist perimeters, and Charlson index for each sex
Guadalupe-Grau et al, 2017 (56)	Spain	Toledo Study for Healthy Aging	Prospective cohort	5.5	M	NM	566	TT, DHEA, DHEAS, E2	110	—	—	—	Age, PASE, hip and waist perimeters, and Charlson index for each sex
Goodman-Gruen et al, 1995 (54)	USA	Rancho Bernardo study	Prospective cohort	19	F	66.7 ± 7.1	624	SHBG	—	153	—	—	Age, BP, TC, HDL, fasting plasma glucose, smoking, alcohol, BMI
Goodman-Gruen et al, 1995 (54)	USA	Rancho Bernardo study	Prospective cohort	19	M	65.8 ± 7.2	760	SHBG	—	235	—	—	Age, BP, TC, HDL, fasting plasma glucose, smoking, alcohol, BMI
Gyawali et al, 2019 (63)	Australia	MAILES	Prospective cohort	4.9	M	54.2 ± 11.1	1492	TT, SHBG	—	56	—	—	Age, sex, smoking, BMI, IHD, preexisting major diseases, disability, frailty, Geriatric, Depression Scale, the Mini-Mental State Examination, high serum CRP
Haring et al, 2010 (64)	Germany	SHIP	Prospective cohort	7.2	M	20-79	1954	TT	—	—	73	40	Age, WC, smoking, high-risk alcohol use, PA, renal insufficiency, and DHEAS
Holmboe et al, 2015 (11)	Denmark	MONICA I–III and Inter99	Prospective cohort	18.5	M	30-70	5323	TT, FT, SHBG, E2	1533	374	480	—	BMI, study, alcohol consumption, PA
Hyde et al, 2011 (65)	Australia	Health in Men Study	Prospective cohort	5.1	M	70-88	3637	SHBG	—	160	—	—	Age, WHR, prevalent IHD, HTN, dyslipidemia, smoking status, DM, Charlson's weighted comorbidity index
Haring et al, 2012 (66)	Germany	SHIP	Prospective cohort	10	M	52 (37.4, 65.5)	2039	TT	321	157	—	—	Age, BMI, smoking status, exercise, alcohol consumption, dietary pattern, education level
Haring et al, 2013 (50)	USA	Framingham Heart Study	Prospective cohort	10	M	75.5 ± 5.4	254	TT, DHEAS, E2	104	—	—	—	Age, BMI, smoking, TC, HDL, DM, SBP, antihypertensive medication
Hyde et al, 2012 (51)	Australia	Health In Men Study	Prospective cohort	5.1	M	70-88	3637	TT, FT, SHBG	605	207	231	—	Age, WHR, HTN, dyslipidemia, DM, smoking status, Charlson's weighted comorbidity index, prevalent cardiovascular disease, number of cancer diagnoses
Islam et al, 2022 (67)	Australia	ASPREE trial	Follow-up of a clinical trial	4.6	F	74 (71.7, 77.7)	5535	TT, DHEA, SHBG	200	—	—	—	Age, BMI, smoking status, alcohol consumption, DM, dyslipidemia, HTN, impaired renal function, treatment allocation (aspirin vs placebo)
Jia et al, 2020 (23)	USA	ARIC study	Prospective cohort	18	M	62.8 ± 5.7	3650	DHEAS	1402	624	—	—	Age, race, TC, HDL, SBP, antihypertensive medication use, current smoking, DM, BMI, lipid-lowering medication use, eGFR, testosterone, SHBG, plus NT-proBNP
Jia et al, 2020 (23)	USA	ARIC study	Prospective cohort	18	F	74.5 ± 5.1	4042	DHEAS	1420	639	—	—	Age, race, TC, HDL, SBP, antihypertensive medication use, current smoking, DM, BMI, lipid-lowering medication use, estimated glomerular filtration rate, testosterone, and SHBG, plus NT-proBNP
Kalme et al, 2005 (15)	Finland	Seven Countries Study	Prospective cohort	8	M	70-89	335	SHBG	NM	NM	—	—	Age, BMI
Khaw et al, 2007 (9)	UK	EPIC-Norfolk	Nested case-control*^d^*	7	M	42-78	2314	TT	825	369	304	—	Age, date of visit, BMI, SBP, TC, cigarette smoking, DM, alcohol intake, PA, social class, education, DHEAS, androstanediol glucuronide, SHBG
Lopez et al, 2022 (68)	USA	NHANES (1988–1991,1999-2004,2011-2014)	Prospective cohort	7.6	M	46.64	5379	TT, FT	666	160	156		Age, race, and ethnicity, smoking status, history of HTN, PA, alcohol consumption, BMI, sex steroid hormones, hypercholesterolemia
Laouali et al, 2020 (69)	France	Three-City study	Prospective cohort	12	M	> 65	338	TT, BT	—	30	45	55	Center, smoking, alcohol drinking, education level, history of CV disease, HTN, hypercholesterolemia, DM, BMI
Laouali et al, 2018 (70)	France	Three-City study	Prospective cohort	12	M	73.6 ± 5.1	444	TT, BT	166	—	—	—	Mass index, sex steroid hormones, hypercholesterolemia
Laouali et al, 2018 (71)	France	Three-City study	Prospective cohort	12	M	73.9 ± 5.4	472	E2	183	44	57	82	Age, date of visit, BMI, WHR, SBP, cholesterol, history of DM, history of HTN, history of high TC, aspirin use, alcohol intake, cigarette smoking status, PA, social class, education level, SHBG, DHEAS, androstanediol glucuronide
Laughlin et al, 2008 (72)	USA	Rancho Bernardo study	Prospective cohort	11.8	M	63.6-78.9	794	TT, BT	538	—	—	—	Age, BMI, WHR, alcohol use, current smoking, exercise
Lin et al, 2011 (49)	USA	NHANES III (1988-1991)	Prospective cohort	15.6	M		409	FT	152	33	NM	NM	Age, cigarette smoking
Li et al, 2024 (73)	China	GBCS	Prospective cohort	10.5	M	66.9 ± 6.7	3948	TT, FT, BT, SHBG	949	312	—	—	Age, education, occupation, personal annual income, smoking status, PA, BMI, hypertension, dyslipidemia, diabetes, and self-reported cardiovascular disease (except for cardiovascular disease mortality)
Menke et al, 2010 (74)	USA	NHANES (1988-1991)	Prospective cohort	16	M	40	1114	E2, BT, SHBG	NM	—	—	—	Age, race-ethnicity, smoking status, pack-years of smoking, household income, education, alcohol consumption, exercise, percent body fat, testosterone, estradiol, SHBG
Maggio et al, 2009 (75)	Italy	In-CHIANTI	Prospective cohort	9	F	65-102	5009	E2	135	—	—	—	Age, WHR, CRP, education, cognitive function, PA, caloric intake, smoking, DM, cardiovascular disease, cancer, COPD, TT
Maggio et al, 2007 (76)	Italy	In-CHIANTI	Prospective cohort	6	M	65-92	410	BT, DHEAS	126	—	—	—	Age, BMI, cancer, interleukin 6, education, cognitive function, depression, PA, caloric and alcohol intake, smoking, coronary heart disease (angina and MI), congestive heart failure, stroke, DM, HTN, Parkinson disease, peripheral artery disease, asthma, cancer, COPD
Mukama et al, 2023 (77)	Germany	EPIC-Heidelberg	Nested case cohort	11.7	M	40.3-65.4	1607	DHEAS	314	96	125	—	Age at blood draw, time at blood collection, smoking status, BMI, waist-to-hip ratio, PA, self-reported diabetes
Mukama et al, 2023 (77)	Germany	EPIC-Heidelberg	Nested case cohort	12.2	F	35.2-66	800	DHEAS	151	38	73	—	Age at blood draw, time at blood collection, smoking status, BMI, waist-to-hip ratio, PA, self-reported diabetes, use of oral contraceptives at blood collection
Ohlsson et al, 2010 (21)	Sweden	MrOS Sweden cohort	Prospective cohort	4.5	M	75.5 ± 3.2	2644	DHEA, DHEAS	328	—	—	—	Age
Pye et al, 2014 (78)	Multiple countries^*c*^	European Male Aging Study	Prospective cohort	4.3	M	59.49 ± 10.48	2599	TT, FT	147	56	—	—	Age, center, BMI, current smoking, poor general health
Szulc et al, 2009 (79)	France	MINOS	Prospective cohort	10	M	50-85	782	TT	182	—	—	—	Age, BMI, smoking, PA, physical performance, health status, vitamin D supplementation
Srinath et al, 2015 (80)	USA	ARIC study	Prospective cohort	12.8	M	63.1 ± 5.6	1588	TT	347	92	—	—	Age, race/center, BMI, WC, cigarette smoking, DM, HTN, LDL, and HDL
Shores et al, 2014 (81)	USA	Cardiovascular Health Study	Prospective cohort	8.9	M	76	1032	TT, FT	777	—	—	—	Age, race, clinic site, smoking status, alcohol consumption, SBP, antihypertensive use, HDL, BMI, WC, SHBG
Schaffrath et al, 2015 (16)	Germany	SHIP	Prospective cohort	10.9	F	49 ± 16.1	1638 (TT), 1525 (FT), 1970 (SHBG)	TT, FT, SHBG	NM	NM	—	—	Age, smoking status, WC, PA, alcohol consumption
Sievers et al, 2010 (12)	Germany	DETECT study	Prospective cohort	4.5	F	57.96 ± 14.37	2914	TT	103	—	—	—	Age, BMI, smoking
Schederecker et al, 2020 (48)	Germany	KORA-F4	Prospective cohort	8.7	M	61.65	1006	SHBG, TT, FT	128	60	34	34	Age, batch, SBP, LDL, lipid-lowering medication, smoking status, antihypertensive medication, BMI, education years, PA, alcohol consumption, eGFR, HsCRP prevalent cancer/ myocardial infarction/ stroke/DM, TT, E2
Schederecker et al, 2020 (48)	Germany	KORA-F4	Prospective cohort	8.7	F	63.08	709	SHBG, TT, FT	70	25	26	19	Age, batch, SBP, LDL, lipid-lowering medication, smoking status, antihypertensive medication, BMI, education years, PA, alcohol consumption, eGFR, HsCRP prevalent cancer/ myocardial infarction/ stroke/DM, TT, E2
Smith et al, 2005 (55)	UK	Caerphilly Study	Prospective cohort	16.5	M	45-59	2515	TT	482	192	—	290	Smoking status, adult social class, alcohol consumption, height, FEV1/height^2^, fibrinogen, white cell count, SBP, TG, TC, HDL BMI, FPG, fasting insulin
Trivedi et al, 2001 (25)	England	Cambridge General Practice Study	Prospective cohort	7.4	M	65-76	963	DHEAS	117	104	—	—	Age, BP, BMI, TC, current smokers, steroid use, past history of cardiovascular disease or cancer
Trivedi et al, 2001 (25)	England	Cambridge General Practice Study	Prospective cohort	7.4	F	65-76	1171	DHEAS	119	55	—	—	Age, BP, BMI, TC, current smokers, steroid use, past history of cardiovascular disease or cancer
Tivesten et al, 2009 (10)	Sweden	MrOS Sweden cohort	Prospective cohort	4.5	M	75.4 ± 3.2	2639 (TT, E2), 2618 (FT), 2625 (SHBG)	TT, FT, E2, SHBG	383	—	—	—	Age, MrOS site, BMI, PA, current smoking
Vikan et al, 2009 (82)	Norway	Tromsø Study	Prospective cohort	11.2 (All-cause), 10 (CVD)	M	59 ± 10.2	1568	TT, E2, FT	395	130	—	—	Age, SBP, HDL/TC, self-reported DM, current smoking, WHR
Wang et al, 2021 (14)	UK	UK Biobank	Prospective cohort	8.9	F	≥ 30	93314	TT, FT, SHBG	2435	346	1583	506	Age at blood draw, ethnicity, fasting status, college or university degree, BMI, smoking status, alcohol consumption, summed metabolic equivalent of task-hours per week for all activity, family history of CVD or cancer, FPG, HDL, LDL, hormone replacement therapy, SHBG
Wang et al, 2021 (14)	UK	UK Biobank	Prospective cohort	8.9	M	≥ 30	154962	TT, FT, SHBG	5754	1243	2987	1524	Age at blood draw, ethnicity, fasting status; college or university degree, BMI, smoking status, alcohol consumption, summed metabolic equivalent of task-hours per week for all activity, family history of CVD or cancer, FPG, HDL, LDL, TT
Yeap et al, 2014 (83)	Australia	Health In Men Study	Prospective cohort	6.7	M	70-89	3690	TT, FT, E2	974	325	—	—	Age, education, smoking, BMI, WHR, HTN, dyslipidemia, DM, creatinine, prevalent CVD, cancer
Zeller et al, 2019 (13)	Finland	FINRISK97 study	Prospective cohort	13.8	F	46.9 ± 21	3961	TT	269	—	—	—	Age, geographical region, TC, HDL, SBP, known HTN, known DM, smoking status, time period of blood drawn
Zeller et al, 2019 (13)	Finland	FINRISK97 study	Prospective cohort	13.8	M	48.2 ± 22.6	3710	TT	510	—	—	—	Age, geographical region, TC, HDL, SBP, known HTN, known DM, smoking status, time period of blood drawn

Abbreviations: ARIC, Atherosclerosis Risk in Communities study; BMI, body mass index; BP, blood pressure; BT, bioavailable testosterone; CRP, C-reactive protein; DETECT, Diabetes Cardiovascular Risk-Evaluation, Targets and Essential Data for Commitment of Treatment; DHEA, dehydroepiandrosterone; DHEAS, dehydroepiandrosterone sulfate; DM, diabetes; E2, estradiol; eGFR, estimated glomerular filtration rate; FPG, fasting plasma glucose; FT, free testosterone; HDL, high-density lipoprotein cholesterol; HTN, hypertension; IHD, ischemic heart diseases; LDL, low-density lipoprotein cholesterol; MAILES, Men Androgen Inflammation Lifestyle Environment and Stress cohort study; MI, myocardial infarction; MrOS, Swedish Osteoporotic Fractures in Men; NHANES, National Health and Nutrition Examination Survey; NM, Not mentioned; NT-proBNP, N-terminal pro-B-type natriuretic peptide; PA, physical activity; PACE, physical activity scale for elderly; SBP, systolic blood pressure; SHBG, sex hormone-binding globulin; SHIP, Study of Health in Pomerania; TC, total cholesterol; TG, triglycerides; TT, total testosterone; WC, waist circumferences; WHR, waist/hip ratio.

^*a*^Follow-up time is reported in years.

^*b*^Age is presented as minimum and/or maximum, mean ± standard deviation, or median (range).

^*c*^Florence, Italy; Leuven, Belgium; Malmö, Sweden; Manchester, United Kingdom; Santiago de Compostela, Spain; Lódz, Poland; Szeged, Hungary; Tartu, Estonia.

^*d*^Prospective population study.

### Meta-Analyses and Dose-Response Analyses

**Figure 2.**
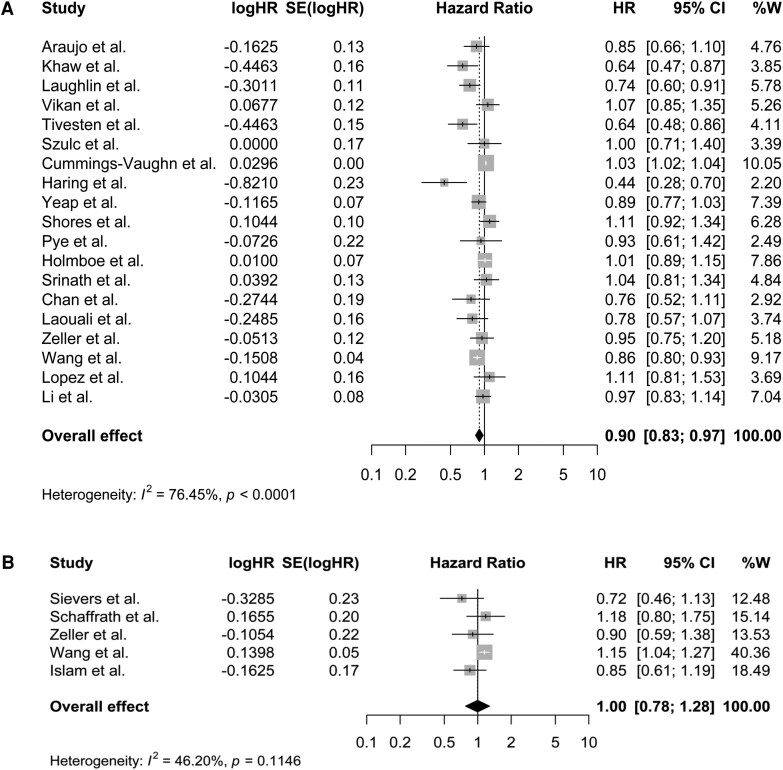
Summary hazard ratios of all-cause mortality for top vs bottom third of total testosterone (TT) levels in (A) men and (B) women.

**Figure 3.**
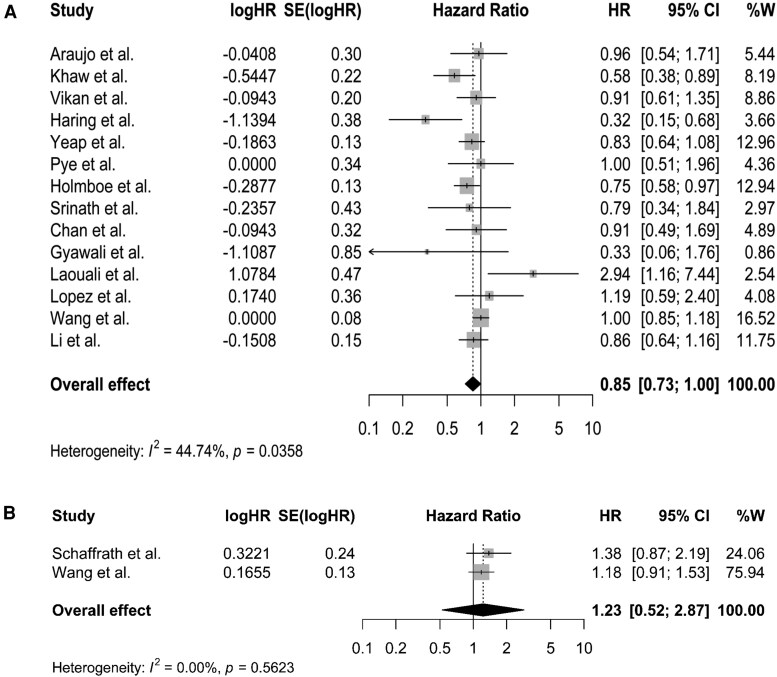
Summary hazard ratios of CVD mortality for top vs bottom third of total testosterone (TT) levels in (A) men and (B) women.

**Figure 4.**
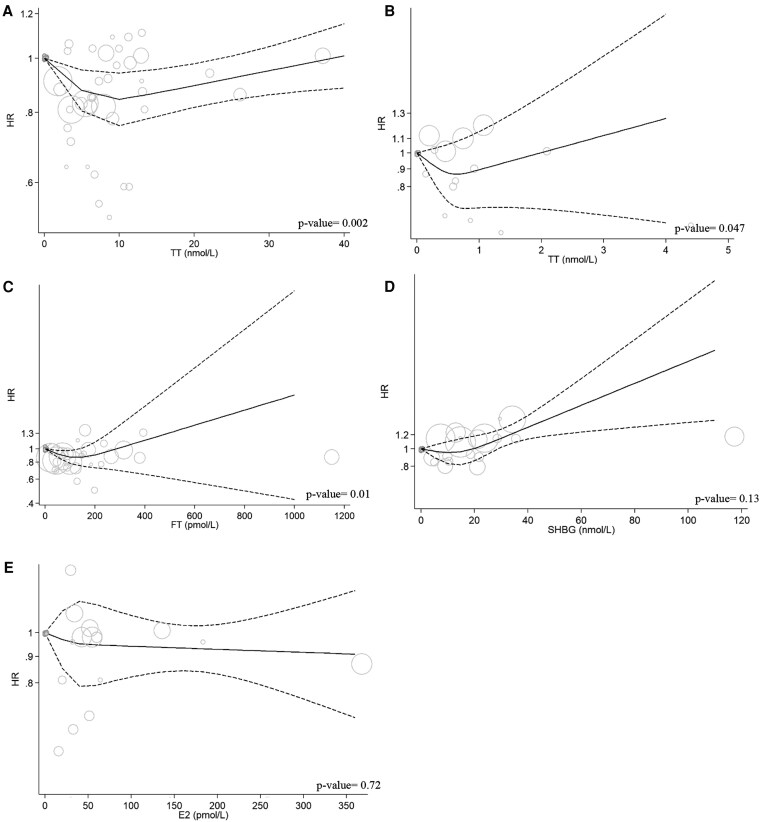
Nonlinear dose-response association of sex hormones and all-cause mortality. (A) TT-Men; (B) TT-Women; (C) FT-Men; (D) SHBG-Men; (E) E2-Men.

A summary of the hazard ratios of mortality outcomes for top vs bottom third of each of the endogenous sex steroid hormones and SHBG are presented in Figs. 2 and 3 and Supplementary Figs. S1 to S4 (34). Findings of the dose-response analyses are presented in Fig. 4, Supplementary Figs. S10 to S12 (34), and Supplementary Table S4 (34). The GRADE evidence for these associations is summarized in Supplementary Tables S5 to S8 (34).

#### Total testosterone

Our meta-analyses showed a lower risk of all-cause mortality in the top vs bottom third of TT in men (HR [95% CI]: 0.90 [0.83 to 0.97], n = 19, *I^2^*: 76.45%, Fig. 2A) but not in women (HR (95% CI): 1.00 [0.78 to 1.28], n = 5, *I^2^*: 46.20%, Fig. 2B). Nonlinear dose-response analysis suggested a U-shaped association in men (*P*_nonlinearity_: 0.002, Fig. 4A) and a similar but slightly more J-shaped association in women (*P*_nonlinearity_: 0.047, Fig. 4B). The certainty of evidence was graded as low for men and high for women (Supplementary Table S6 and Supplementary Table S7) (34).

No association was observed between TT levels and risk of CVD mortality in either men (HR [95% CI]: 0.85 [0.71 to 1.00], *I^2^*: 44.74%, n = 14, Fig. 3A) or women (HR [95% CI]: 1.23 [0.52 to 2.87], n = 2, Fig. 3B). The certainty of evidence was low for men and moderate for women (Supplementary Table S6 and Supplementary Table S7) (34). For cancer mortality or death due to the other causes, studies were only available to be pooled in men, and these showed a lower risk of mortality due to other causes (HR [95% CI]: 0.82 0.70 to 0.94], *I^2^*: 0.0%, n = 4, Supplementary Fig. S4A) (34), with a high certainty of evidence for this association (Supplementary Table S6**)** (34) and no statistically significant association for risk of cancer mortality (HR [95% CI]: 0.90 [0.68 to 1.18], *I^2^*: 72.6%, n = 7, Supplementary Fig. S3A) (34), with a low certainty of evidence (Supplementary Table S6) (34). However, only one study by Wang et al reported the association of TT and risk of cancer or other causes mortality in women (Table 1).

#### Free testosterone

We observed no association between top vs bottom third of FT level and risk of all-cause mortality in men (HR [95% CI]: 0.94 [0.83 to 1.07], *I^2^*: 80.57%, n = 11, Supplementary Fig. S1A) (34) or women (HR [95% CI]: 1.04 [0.93 to 1.17], n = 2, Supplementary Fig. S1B) (34). The dose-response analysis was only feasible in men and showed a J-shaped association (*P*_nonlinearity_: 0.01, Fig. 4C). The certainty of evidence was moderate for men and high for women (Supplementary Table S6 and Supplementary Table S7) (34). Similarly, pooled HR showed no association with risk of CVD mortality in either men (HR [95% CI]: 1.04 [0.83 to 1.30], n = 9, *I^2^*: 70.48%, Supplementary Fig. S2A) (34) or women (HR [95% CI]: 1.27 [0.23 to 6.86], n = 2, Supplementary Fig. S2B) (34). The certainty of evidence was very low for men and moderate for women (Supplementary Table S6 and Supplementary Table S7) (34).

The analysis in men showed no association with risk of cancer mortality (HR [95% CI]: 0.94 [0.76 to 1.16], *I^2^*: 17.42%, n = 4, Supplementary Fig. S3B) (34) and mortality due to the other causes (HR [95% CI]: 0.80 [0.19 to 3.29), *I^2^*: 71.5%, n = 2, Supplementary Fig. S4B] (34). The certainty of evidence for these associations was moderate (Supplementary Table S6) (34).

Only one study, by Wang et al, reported the association of FT and risk of cancer and other causes mortality in women (Table 1).

#### Bioavailable testosterone

Our analysis showed no association between level of BT with risk of all-cause mortality (HR [95% CI]: 0.93 ([0.80 to 1.07], *I^2^*: 61.28%, n = 6, Supplementary Fig. S1C) (34) in men with moderate certainty of evidence (Supplementary Table S6) (34) and CVD mortality (HR [95% CI]: 1.56 [0.69 to 480.12], *I^2^*: 76.30%, n = 2, Supplementary Fig. S2C) (34) in this population with low certainty of evidence (Supplementary Table S6) (34).

One study, by Laouali et al, reported the association of BT and risk of cancer and other causes of mortality in men (Table 1).

#### Sex hormone-binding globulin

Our meta-analysis showed no association between SHBG and risk of all-cause mortality in men (HR [95% CI]: 1.10 [0.94 to 1.28], *I^2^*: 80.46%, n = 8, Supplementary Fig. S1D) (34) but a higher risk in women (HR [95% CI]: 1.25 [1.13 to 1.39], *I^2^*: 0.0%, n = 3, Supplementary Fig. S1E) (34). For men, the certainty of evidence was low while it was high for women (Supplementary Table S6 and Supplementary Table S7) (34). No association was observed for risk of CVD mortality either in men (HR [95% CI]: 0.93 [0.67 to 1.29], *I^2^*: 80.3%, n = 6, Supplementary Fig. S2D) (34) or women (HR [95% CI]: 1.02 [0.75 to 1.39], *I^2^*: 0.0%, n = 2, Supplementary Fig. S2E) (34). The certainty of evidence was moderate for men and high for women (Supplementary Table S6 and Supplementary Table S7) (34). Additionally, no association with the risk of death due to cancer in men was observed (HR [95% CI]: 1.21 [0.99 to 1.48], *I^2^*: 0.0%, n = 3, Supplementary Fig. S3C) (34), with a moderate certainty of evidence (Supplementary Table S5) (34).

One study, by Wang et al, presented the association of SHBG with risk of cancer and other causes of mortality in women (Table 1).

#### Estradiol

Studies examined the association between E2 and risk of all-cause and cause-specific mortality in men and only one study, by Maggio et al, investigated this association in women (Table 1). Pooled effect estimates indicated no association between the top vs the bottom third of E2 and risk of all-cause mortality (HR [95% CI]: 0.99 [0.88 to 1.11], n = 9, *I^2^*: 48.09%, Supplementary Fig. S1F) (34) with a moderate certainty of evidence (Supplementary Table S6) (34), CVD mortality (HR [95% CI]: 1.02 [0.75 to 1.39], n = 6, *I^2^*: 53.64%, Supplementary Fig. S2F) (34) with a low certainty of evidence (Supplementary Table S6) (34), with risk of death due to the other causes (HR [95% CI]: 0.93 [0.68 to 1.28), *I^2^*: 0.0%, n = 3, Supplementary Fig. S4C) (34) with low certainty of evidence in men (Supplementary Table S6) (34), or cancer mortality (HR [95% CI]: 1.28 [0.97 to 1.68], *I^2^*: 0.0%, n = 3, Supplementary Fig. S3D) (34) with a low certainty of evidence (Supplementary Table S6) (34).

#### Dehydroepiandrosterone sulfate and dehydroepiandrosterone

We observed a significant association between the top vs the bottom third level of DHEAS and lower risk of all-cause mortality in men (HR [95% CI]: 0.72 [0.57 to 0.91], *I^2^*: 73.99%, n = 6, Supplementary Fig. S1G) (34) but not in women (HR [95% CI]: 0.84 [0.57 to 1.24], I^2^: 17.03%, n = 3, Supplementary Fig. S1H) (34). The certainty of evidence was very low for men and moderate for women (Supplementary Table S6 and Supplementary Table S7) (34). Three studies reported the association of DHEAS and risk of all-cause mortality in men and women combined; meta-analysis of these studies also showed no association (HR [95% CI]: 0.90 [0.51 to 1.57], Supplementary Fig. S1I) (34) with low certainty of evidence (Supplementary Table S8) (34).

Similarly, we found no association between DHEAS levels and risk of CVD death in men (HR [95% CI]: 0.76 [0.41 to 1.41], *I^2^*: 58.35%, n = 5, Supplementary Fig. S2G) (34) or women (HR [95% CI]: 0.83 [0.52 to 1.30), *I^2^*: 73.47%, n = 4, Supplementary Fig. S2H) (34) with low certainty of evidence (Supplementary Table S6 and Supplementary Table S7) (34).

Pooled effect estimates of 2 studies showed no association between DHEA and risk of all-cause mortality in men (HR [95% CI]: 0.73 [0.12 to 4.55), Supplementary Fig. S1J) (34).

Only one study, by Mukama et al, presented the association of DHEAS and risk of cancer mortality in men and women. One study by Islam et al reported the association of DHEA and all-cause mortality in women and another study by Chan et al reported the association of DHEA and risk of CVD mortality in men (Table 1).

### Additional Analyses

Findings of additional analyses including leave-one-out, publication bias, and subgroup analysis are reported in Supplementary Figs. S5 to S9, Supplementary Table S4, and Supplementary Information 3 (34).

## Discussion

To the best of our knowledge, this study is the first comprehensive systematic review and meta-analysis that also implemented a dose-response analysis on the association between endogenous sex steroid hormones and SHBG with risk of all-cause and cause-specific mortality. Our analysis indicated a 10% lower risk of all-cause mortality and an 18% lower risk of death due to other causes for the top vs the bottom third of TT levels in men, while no associations were observed in women. Also, SHBG levels were associated with an increased risk of all-cause mortality by 25% in women. We found a lower risk of all-cause mortality for the top vs the bottom third of DHEAS level in men, while no associations were seen in women. We only found an association between E2 levels and higher risk of cancer mortality in men and no studies were available in women.

We found a U-shaped association between TT levels and risk of all-cause mortality in men, and a J-shaped association in women. Our findings suggested that while we found no evidence of higher all-cause mortality within increasing TT levels to the highest normal ranges (40 nmol/L) in men, a steeper increase in the risk of all-cause mortality for elevated values of TT in women was observed. Our results challenge the previous concept that lower TT is associated with increased mortality in a linear fashion. A limited number of individual studies have reported the dose-response associations between TT and mortality, which were consistent with our observations. The Swedish Osteoporotic Fractures in Men study (MrOS), including 3104 men with 4.5 years of follow-up, found that men with TT in the second quartile vs the fourth quartile had a lower risk of mortality than those with TT in the first quartile (10). Findings of another study, by Yeap et al (83), including 3690 men with a median follow-up of 6.7 years, showed the lowest risk of mortality with an optimal range of TT (9.8-15.8 nmol/L), which is close to our results (5-15 nmol/L).

A previous meta-analysis on the association of testosterone levels and risk of mortality in men, published more than 10 years ago and based on 11 studies (27), revealed that low endogenous TT levels were associated with an increased risk of all-cause mortality, in line with our results.

A recent individual participant data (IPD) meta-analysis of 9 studies in men investigated the association between testosterone and related hormones and the risk of mortality. Owing to the nature of the study, the authors applied more rigorous eligibility criteria, including community-dwelling men with sex hormone levels measured using mass spectrometry and at least 5 years of follow-up. They accounted for a comprehensive set of potential confounders in their statistical models, including age, body mass index, marital status, alcohol consumption, smoking, physical activity, hypertension, diabetes, creatinine concentration, the ratio of total to high-density lipoprotein cholesterol, and lipid-lowering medication use. They found no association between low vs high levels of TT and risk of all-cause or CVD mortality (84). Our meta-analysis complements the IPD meta-analysis by Yeap et al (84) by applying broader eligibility criteria, including studies on women, additional sex hormones such as DHEA and DHEAS, and a wider range of mortality outcomes, including cancer and other causes.

The results of another more recent systematic review and meta-analysis of observational studies among men indicated a higher risk of overall mortality and CVD mortality with lower TT levels (28). However, this review suffers from methodological issues, for example: by pooling results of different patient groups; not including certain eligible studies performed on general population (11, 61, 83); and pooling the lowest vs highest estimates such as quintile, quartile, and tertile without standardizing them first. Neither of the above-mentioned aggregated meta-analyses evaluated the shape of the dose-response associations nor summarized the associations of BT and FT with the risk of mortality (27, 28).

Most of the evidence regarding the potential protective effects of testosterone has been attributed to its effect on the cardiovascular system. For instance, a large body of literature suggests a direct vasodilator effect of TT on many vascular districts, including the coronary arteries, in addition to a role in endothelium repair (85). Furthermore, studies carried out in hypogonadal subjects demonstrated that testosterone therapy is able to decrease the production of inflammatory cytokines, and to increase the level of the anti-atherogenic cytokine interleukin-10 (86). It has been shown that testosterone replacement therapy decreased the occurrence of typical atherosclerotic processes such as neointima formation and fatty streak accumulation (87). Strong evidence supports it that androgens could decrease arterial stiffness probably by inhibiting vascular smooth muscle calcification (88).

For women, the association of endogenous sex steroid hormones and risk of all-cause and cause-specific mortality has been poorly investigated. Many available studies have been performed on women with a wide age range without any information on the menopausal status and had relatively small sample sizes (12, 13, 16), leading to conflicting results. However, among the included studies on women, only a recent study done by Wang et al (14) investigated the association of endogenous sex steroid hormones with the risk of all-cause and cause-specific mortality on a large number of postmenopausal women (93 314 individuals). This study found a higher risk of all-cause mortality with elevated TT levels in women. Consistent with our findings, the results of a cohort study with a long-term follow-up (up to 30 years) and a relatively large sample size including 4716 women, not receiving oral contraceptives or hormonal replacement therapy, found that extremely high concentrations of TT were associated with increased risk of mortality (52). It is worth mentioning that testosterone levels can be aromatized to E2 in endothelial cells in women (89). Thus, it seems an important factor which needs to be controlled to explore an unbiased association between testosterone levels and mortality. Almost none of the included studies in the current meta-analysis have adjusted their models based on E2 levels.

According to our results, higher SHBG levels were associated with an increased risk of cancer mortality in men and all-cause mortality in women which were independent of free or bioavailable testosterone levels. Consistent with the IPD meta-analysis by Yeap et al (84), we observed a similar direction of association between SHBG levels and the risk of all-cause mortality in men; however, our results did not reach statistical significance. In line with our observation, there is evidence supporting the direct effect of SHBG independent of sex steroids (90, 91). It has been shown that SHBG may mediate cell-surface signaling, cellular delivery, and biological action of endogenous sex steroid hormones via activation of a specific plasma receptor directly (92, 93). Among the included studies, only the study done by Wang et al adjusted their models for TT levels and found increased risk of all-cause mortality with higher SHBG levels in both men and women (14).

In agreement with most of the previous literature, our results were indicative of no association of E2 with risk of all-cause, CVD, and other causes of mortality in men. In addition, findings from a recent IPD meta-analysis reported no association between E2 levels and the risk of all-cause mortality, CVD mortality, or incident CVD events (84). This is consistent with other evidence which showed that E2 was not associated with prevalent CVD or intermittent claudication (94, 95). Findings of a meta-analysis on the association of E2 and CVD-related outcomes in men failed to establish any associations (96). Although we found an increased risk of cancer mortality for the top vs bottom third of E2 levels in men, these findings should be interpreted with caution due to the limited number of studies, low sample size, and lack of having cancer-specific mortality and various covariates in the statistical models. Studies on the association of E2 and risk of mortality in women are lacking and more evidence is needed to explore these associations.

We found a protective association of DHEAS, but not DHEA (top vs bottom third of distribution) with the risk of all-cause mortality in men. Evidence regarding the association of DHEA and DHEAS and risk of mortality is limited and conflicting. Some studies were in support of our findings (21-23), while others found no associations (25, 61). Physiological functions of DHEA/DHEAS in CVD as well as noncancer and non-CVD disease defense may justify these findings. DHEA(S) has been shown to be an effective activator of peroxisome proliferator-activated receptor-α (97, 98), which may contribute as a modulator of immune functions (99, 100) and oxidative stress (97) as well as atherosclerosis (101). Moreover, low DHEA(S) has been recognized as general marker of poor health, hence an epiphenomenon of subclinical diseases (98).

### Strengths and Limitations

We included all evidence on the association of endogenous sex steroid hormones and SHBG with the risk of all-cause and cause-specific mortality and reported the sex-specific associations, which has not been done before. In order to have a consistent approach to conducting the meta-analysis, we transformed the estimates, which often were reported differently by each study (such as per unit or per 1-SD change or comparing quintiles, quartiles, tertiles, etc.), to the top vs bottom third of endogenous sex steroid hormones distribution, which is applied by the previous meta-analysis concerning the association of biomarkers and risk of chronic diseases and mortality (102-104).

Residual confounding is inevitable and should be considered due to the nature of the included study designs (observational cohort studies). We pooled the results of multiple cohort studies with different methods of hormonal assessment, sample sizes, follow-up duration, and adjustments in the statistical methods; however, an IPD meta-analysis might provide more accurate evidence regarding these associations.

While we included the studies on the general population, the bias due to the preexisting diseases might stand. Also, our study explores only baseline values of endogenous sex steroid hormones and SHBG with mortality, future research could benefit from incorporating longitudinal, repeated measurements of sex hormones to better understand how changes in these levels over time may influence mortality risk.

### Policy Implications and Future Research

Future studies, such as large prospective cohort studies or clinical trials, should explore dose-response associations and identify sex-specific cutoff levels of endogenous sex steroid hormones and SHBG that could be critical for both men and women.

Our findings also highlight a gap in understanding the impact of endogenous sex steroid hormones on mortality in different sexes, considering most of the studies were based in male individuals. Our study also provides potential support for future studies such as Mendelian randomization and clinical trials to explore causality separately in men and women. Most included studies investigated the association of endogenous sex steroid hormones and all-cause and CVD mortality and only a limited number of studies have investigated the association of endogenous sex steroid hormones with cancer mortality in both sexes. Sex differences in incidence and mortality of cancer have been highlighted in the last decades (105, 106). Growing evidence shows sex-specific differences in the incidence and mortality of cancers not only for sex-specific organs such as prostate and colorectal cancers in males and breast cancer in females, but also for colon, lung, and liver cancers (107, 108). Overall, cancer mortality is greater in men than in women with lung, colorectal, and stomach cancers as the leading causes of mortality in men rather than women (107). Thus, future studies on the association between these hormones and the risk of specific cancer mortality may provide more evidence of these sex differences. Additionally, considering the function and relationship between steroid hormones and hormones such as luteinizing hormone (LH) or other factors such as hematocrit (Hct), exploring the association between steroid hormones and the risk of death while accounting for LH and Hct would add valuable insights. LH, as a key regulator of testosterone, could serve as a marker of hormonal dysregulation, while Hct, influenced by testosterone (109), may affect risk of cardiovascular diseases (110-113). Investigating these interactions could enhance our understanding of the mechanisms linking steroid hormones to mortality.

Most of the studies included in the current analysis were from North America, Europe, and Australia and evidence on this topic is lacking for some ethnic groups such as Africans and Asians. Since racial/ethnic differences have been proposed for endogenous sex steroid hormones for either men or women, more studies are needed to be performed on other ethnic groups (114, 115).

Among the included studies only 3 studies adjusted their statistical models for SHBG (9, 14, 81). As a major carrier of testosterone and E2, SHBG correlates with TT and E2 levels and has been associated with a higher risk of all-cause mortality. Thus, the complex relations between androgens, SHBG, and mortality needs further investigation.

It should be noted that sex steroid hormones and SHBG levels were measured using different commercial methods, primarily immunoassays rather than liquid chromatography-tandem mass spectrometry (LCMS). This is particularly important for the measurement of TT in women and E2 in postmenopausal women and men since immunoassays are less reliable due to method-specific bias and inaccuracy in low sex hormone concentrations. Moreover, FT and BT are calculated variables that lack certified standards and are dominated by SHBG. While we aimed to investigate the dose-response association between endogenous sex steroid hormones, SHBG, and mortality risk, these methodological limitations should be considered. Our dose-response findings highlight the need for future studies to move beyond the conventional approach of categorizing sex steroid hormones and SHBG into high or low levels when examining their associations with chronic diseases and mortality. Instead, research should focus on dose-response relationships separately in men and women. Future large individual participant meta-analysis can help explore and define sex hormones cutoffs related to mortality in men and women, which could have clinical and public health implication for introducing prevention strategies.

## Conclusion

Our findings provide more evidence on the sex-specific associations of endogenous sex steroid hormones and SHBG with risk of all-cause and cause-specific mortality, which may help to implement sex-specific randomized clinical trials of sex steroid hormones treatments to assess whether modulating these hormones might reduce risk of mortality and improve longevity.

## Data Availability

Not applicable.

## PROSPERO Registration

PROSPERO registration code: CRD42022329605.

## Abbreviations

BT: bioavailable testosterone
CVD: cardiovascular disease
DHEA: dehydroepiandrosterone
DHEAS: dehydroepiandrosterone sulfate
E2: estradiol
FT: free testosterone
HR: hazard ratio
IPD: individual participant data
OR: odds ratio
ROBINS-E: Risk Of Bias In Non-randomized Studies of Exposures
SE: standard error
SHBG: sex hormone-binding globulin
TT: total testosterone
